# Genome-wide methylation analysis in Silver–Russell syndrome, Temple syndrome, and Prader–Willi syndrome

**DOI:** 10.1186/s13148-020-00949-8

**Published:** 2020-10-22

**Authors:** Kaori Hara-Isono, Keiko Matsubara, Tomoko Fuke, Kazuki Yamazawa, Kazuhito Satou, Nobuyuki Murakami, Shinji Saitoh, Kazuhiko Nakabayashi, Kenichiro Hata, Tsutomu Ogata, Maki Fukami, Masayo Kagami

**Affiliations:** 1 Department of Molecular Endocrinology, National Research Institute for Child Health and Development, 2-10-1 Okura, Setagaya-ku, Tokyo, 157-8535 Japan; 2 Department of Pediatrics, Keio University School of Medicine, 35 Shinanomachi, Shinjuku-ku, Tokyo, 160-8582 Japan; 3 Medical Genetics Center, National Hospital Organization Tokyo Medical Center, 2-5-1 Higashigaoka, Meguro-ku, Tokyo, 152-8902 Japan; 4 Department of Genome Medicine, National Research Institute for Child Health and Development, 2-10-1 Okura, Setagaya-ku, Tokyo, 157-8535 Japan; 5 Department of Pediatrics, Dokkyo Medical University Saitama Medical Center, 2-1-50 Minami Koshigaya, Koshigaya, 343-8555 Japan; 6 Department of Pediatrics and Neonatology, Nagoya City University Graduate School of Medical Sciences, 1 Kawasumi, Mizuho-cho, Mizuho-ku, Nagoya, 467-8601 Japan; 7 Department of Maternal Fetal Biology, National Research Institute for Child Health and Development, 2-10-1 Okura, Setagaya-ku, Tokyo, 157-8535 Japan; 8 Department of Pediatrics, Hamamatsu University School of Medicine, 1-20-1 Handayama, Higashi-ku, Hamamatsu, 431-3192 Japan

**Keywords:** Imprinting disorders, Genome-wide methylation analysis, HumanMethylation450 BeadChip, Silver–Russell syndrome, Temple syndrome, Prader–Willi syndrome

## Abstract

**Background:**

Imprinting disorders (IDs) show overlapping phenotypes, particularly in Silver–Russell syndrome (SRS), Temple syndrome (TS14), and Prader–Willi syndrome (PWS). These three IDs include fetal and postnatal growth failure, feeding difficulty, and muscular hypotonia as major clinical features. However, the mechanism that causes overlapping phenotypes has not been clarified. To investigate the presence or absence of methylation signatures associated with overlapping phenotypes, we performed genome-wide methylation analysis (GWMA).

**Results:**

GWMA was carried out on 36 patients with three IDs (SRS [*n* = 16], TS14 [*n* = 7], PWS [*n* = 13]) and 11 child controls using HumanMethylation450 BeadChip including 475,000 CpG sites across the human genome. To reveal an aberrantly methylated region shared by SRS, TS14, and PWS groups, we compared genome-wide methylation data of the three groups with those of control subjects. All the identified regions were known as SRS-, TS14-, and PWS-related imprinting-associated differentially methylated regions (iDMRs), and there was no hypermethylated or hypomethylated region shared by different ID groups. To examine the methylation pattern shared by SRS, TS14, and PWS groups, we performed clustering analysis based on GWMA data. The result focusing on 620 probes at the 62 known iDMRs (except for SRS-, TS14-, and PWS-related iDMRs) classified patients into two categories: (1) category A, grossly normal methylation patterns mainly consisting of SRS group patients; and (2) category B, broad and mild hypermethylation patterns mainly consisting of TS14 and PWS group patients. However, we found no obvious relationship between these methylation patterns and phenotypes of patients.

**Conclusions:**

GWMA in three IDs found no methylation signatures shared by SRS, TS14, and PWS groups. Although clustering analysis showed similar mild hypermethylation patterns in TS14 and PWS groups, further study is needed to clarify the effect of methylation patterns on the overlapping phenotypes.

## Background

Imprinting disorders (IDs) are clinical syndromes caused by changes in expression of the imprinted genes. The imprinting-associated differentially methylated region (iDMR) has parental-origin-specific DNA methylation and functions as a regulator for the imprinted gene within a single imprinted region [1]. The iDMRs consist of germline DMRs imprinted during gametogenesis and secondary DMRs imprinted after fertilization, and the methylation pattern of the germline DMR hierarchically regulates that of the secondary DMR within the same imprinted region [1]. Recently, several studies reported the extent of iDMRs examined by genome-wide parent-of-origin methylation analysis using a high-density DNA methylation array [2, 3].

Some IDs have overlapping phenotypes affecting growth, development, and metabolism [4]. Recently, overlapping clinical features among IDs, such as Silver–Russell syndrome (SRS), Temple syndrome (TS14), and Prader–Willi syndrome (PWS), have been reported, regardless of the different imprinted regions affected [5, 6]. The diagnosis of SRS is made based on clinical features. Recently, the Netchine–Harbison clinical scoring system (NH-CSS) which includes prenatal and postnatal growth failure, relative macrocephaly at birth, protruding forehead, feeding difficulty, and body asymmetry, was adopted as the primary clinical diagnostic criteria for SRS [7, 8]. Hypomethylation of the *H19/IGF2*:IG-DMR on chromosome 11p15.5 (*H19*LOM) detected in approximately 50% of SRS patients results in decreased *IGF2* expression and increased *H19* expression [9]. Furthermore, maternal uniparental disomy (UPD) of chromosome 7 (UPD(7)mat) was detected in about 10% of SRS patients [9]. TS14 is characterized by small for gestational age, muscular hypotonia in early infancy, early onset puberty, and markedly short adult stature [10]. TS14 is genetically diagnosed, and the etiologies of TS14 are maternal UPD of chromosome 14 (UPD(14)mat), paternal microdeletions involving the 14q32.2 imprinted region, and epimutation of the paternally derived *MEG3/DLK1*:IG-DMR and *MEG3*:TSS-DMR at 14q32.2 [10]. PWS is characterized by hypotonia and/or feeding difficulty in neonates, behavioral problems, and global developmental delay [11]. PWS results from paternal 15q11-q13 deletion, maternal UPD of chromosome 15 (UPD(15)mat), and epimutation of the *SNURF*/*SNRPN* locus [12]. Growth failure, feeding difficulty, and muscular hypotonia are overlapping features in all these IDs. In particular, the phenotypic spectrum of TS14 is wide and often overlaps with that of SRS and/or PWS, especially in infancy to early childhood. Indeed, 27 out of 32 patients with TS14, who were diagnosed by molecular testing, were initially suspected as having SRS and/or PWS because of their clinical features, such as some NH-CSS features and muscular hypotonia, frequently observed in PWS patients in the neonatal period and early infancy [5]. In this regard, Habib et al. have performed transcriptome analysis using skin fibroblast and/or leukocyte samples obtained from patients with TS14 and SRS with *H19*LOM, showing decreased *IGF2* expression in TS14 and SRS patients and decreased *SNURF* and *IPW* expression, which is the representative expression pattern of PWS, in TS14 patients [6]. This would be relevant to the phenotypic similarities between TS14 and SRS and between TS14 and PWS. However, the exact molecular mechanism for overlapping phenotypes has not been clarified. In addition, some genes including imprinted genes have very weak or no expression in leukocytes and skin fibroblasts, which are easily obtainable tissues from patients.

Genome-wide methylation analysis (GWMA) is a powerful tool for the identification of DNA methylation differences. GWMA reveals associations between DNA methylation patterns and various health problems [13–15]. Recent GWMA studies for SRS patients showed novel methylation changes other than *H19/IGF2*:IG-DMR, for example, hypermethylation of the *RB1*:Int2-DMR [16], hypomethylation of the CpG sites at the *OSBPL5* gene [17], and hypomethylation of the promoter of the *HOXA4* gene [18], although each study detected a separate region. Another GWMA in 65 patients with various IDs identified 23 patients with multilocus imprinting disturbance (MLID) [19], the condition of having abnormally methylated iDMR(s) other than disease-responsible iDMR(s). To our knowledge, there is no study attempting to explain phenotypic similarities among patients with different IDs and phenotypic variations among patients with the same ID, in terms of GWMA-based methylation status.

Here, we report GWMA-based methylation data obtained from 36 patients with SRS, TS14, or PWS. To investigate the presence or absence of methylation signatures associated with overlapping phenotypes, we examined (1) aberrantly methylated regions shared by three IDs and (2) methylation patterns shared by three IDs.

## Results

### Subjects

The total of 36 patients consisted of (1) 16 patients with *H19*LOM (SRS group, all with epimutations) consisting of 14 patients with more than four out of six NH-CSS features and two patients with three out of six NH-CSS features plus triangular face and/or short 5th clinodactyly; (2) seven patients with hypomethylated *MEG3/DLK1*:IG-DMR and *MEG3*:TSS-DMR (TS14 group) consisting of four with UPD(14)mat (TS14 subgroup-1) and three with epimutations (TS14 subgroup-2); and (3) 13 patients with hypermethylated *SNURF*-TSS-DMR (PWS group) consisting of six with UPD(15)mat (PWS subgroup-1), three with epimutation (PWS subgroup-2), and four with deletions (PWS subgroup-3). Twelve patients with SRS [20, 21], seven patients with TS14 [5], and 11 control subjects were previously studied [22]. The genetic cause and clinical characteristics of the patients are shown in Additional file 1: Table S1. We did not include PWS group Pts. 3 and 6 because information remained quite fragmentary.

### GWMA

GWMA was carried out using HumanMethylation450 BeadChip (HM450k) (Illumina, San Diego, CA) featuring approximately 475,000 CpGs across the human genome, except for CpGs which show age-related drift [23–25], sex-bias [26], and striking change before/after puberty [27]. We performed the Crawford–Howell *t* test (CH *t*-test) to evaluate the methylation levels of 809 CpG sites on 77 iDMRs defined by Monk [28] and Joshi [3] in each patient for confirming the abnormal methylation level of each ID-related iDMR, and for detecting MLID. The actual methylation levels (*β* values) of 809 analyzed CpG sites in each patient are shown in Additional file 2: Table S2, together with the ∆*β* of CpGs, the difference between *β* value of each patient and the average *β *value of the control group, and the false discovery rate (FDR) compared to the average *β* value of the control group. (Genome-wide methylation data are not shown.)

**Fig. 1:**
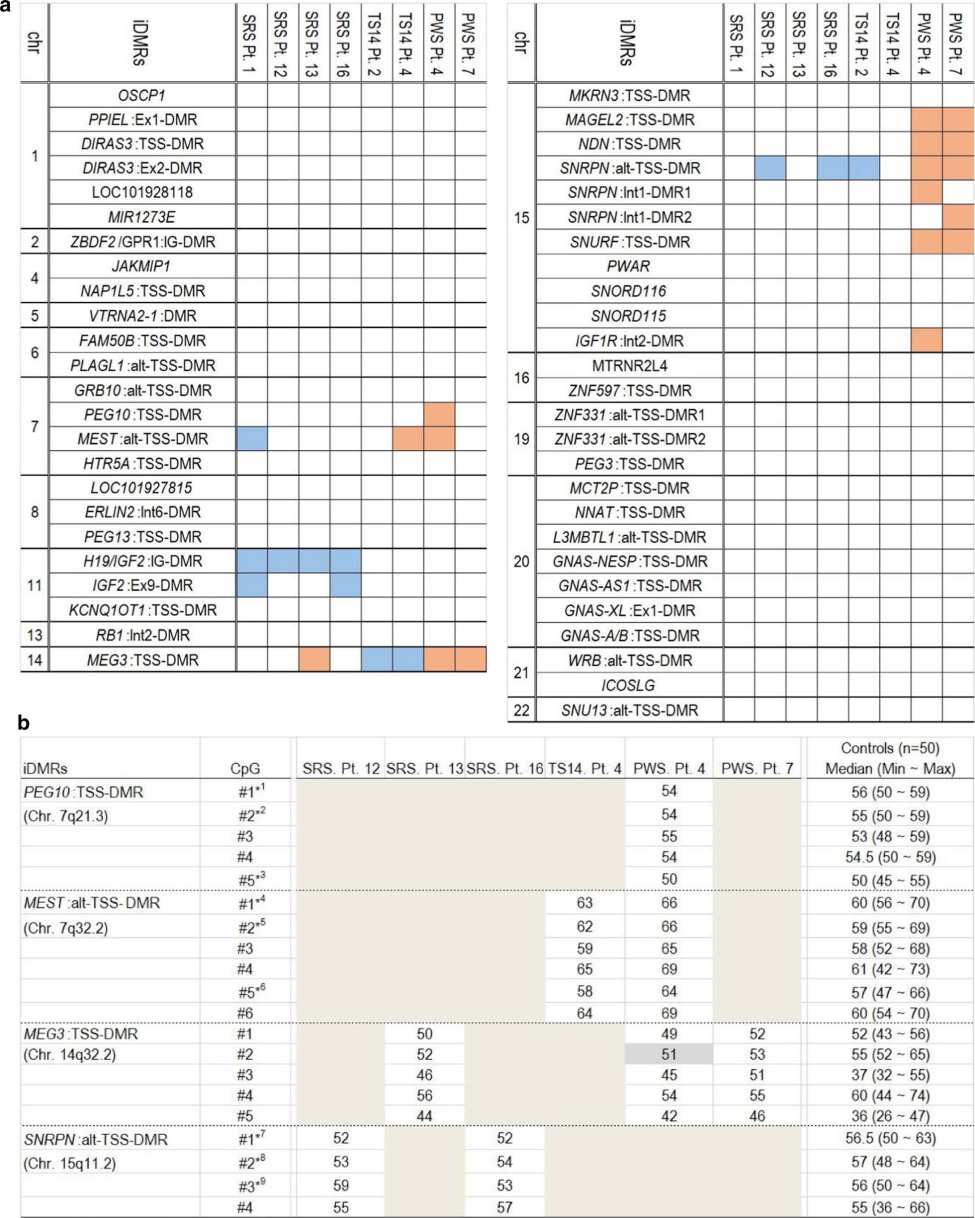
**a** Methylation status of MLID patients focusing on 50 known iDMRs including at least four probes. Red and blue boxes indicate the iDMRs contained more than two consecutive probes showing hypermethylation (red) or hypomethylation (blue). **b** Methylation indices (%) for CpGs at additionally affected iDMRs in each MLID patient determined by pyrosequencing analysis. SRS Pt. 1 and TS14 Pt. 2 are not included due to lack of remaining samples. Median and normal ranges are calculated using the results of 50 healthy controls. The hypomethylated CpG sites are highlighted with light gray backgrounds. *1–9 are CpG sites that have also been examined by the HM450k (^*1^cg19079047, ^*2^cg02965180, ^*3^cg05277165, ^*4^cg19344806, ^*5^cg23714917, ^*6^cg21200654, ^*7^cg11826663, ^*8^cg24785225, ^*9^cg10271763). *chr* chromosome, *iDMRs* imprinting-associated differentially methylated regions, *SRS* Silver–Russell syndrome, *TS14* Temple syndrome, *PWS* Prader–Willi syndrome, *MLID* multilocus imprinting disturbance, *HM450k* HumanMethylation450 BeadChip

We confirmed that all SRS, TS14, and PWS patients in our study showed aberrant methylation levels of the SRS-, TS14-, and PWS-related iDMRs, respectively (Additional file 2: Table S2). In addition, the GWMA data suggested that eight patients had MLID, consisting of four patients in the SRS group, two patients in the TS14 group, and two patients in the PWS group (Fig. 1a). The patients with MLID showed additional affected iDMRs ranging from one to three. Of eight patients with MLID, three patients (TS14 Pts. 2 and 4, PWS Pt. 4) had UPD, one patient (PWS Pt. 7) had deletion, and the remaining patients had epimutations. However, confirmation analysis by pyrosequencing showed normal methylation levels at all additionally affected iDMRs detected by HM450k (Fig. 1b).

#### Aberrantly methylated regions in SRS, TS14, and PWS groups/subgroups

**Fig. 2:**
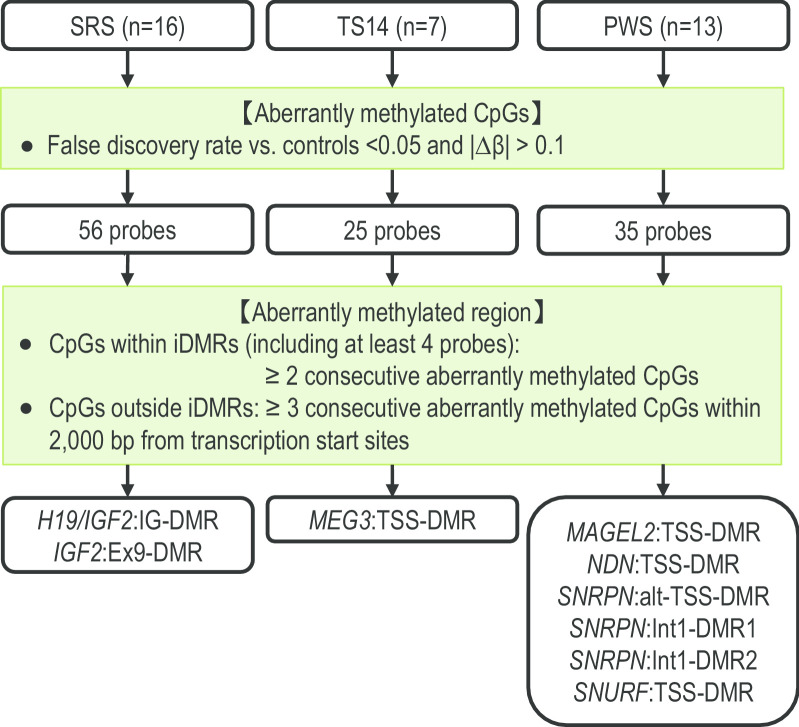
The filtering process for detecting aberrantly methylated regions in SRS, TS14, and PWS groups. Based on the average *β* value of each ID group and controls at each probe site, we extracted aberrantly methylated CpGs and regions. *SRS* Silver–Russell syndrome, *TS14* Temple syndrome, *PWS* Prader–Willi syndrome, *iDMRs* imprinting-associated differentially methylated regions

We attempted to reveal an aberrantly methylated region shared by SRS, TS14, and PWS groups/subgroups. In this regard, we compared the average *β* value of each ID group and controls at each probe site and extracted aberrantly methylated 56, 25, and 35 CpGs as defined in Fig. 2 in SRS, TS14, and PWS groups, respectively. Subsequently, we evaluated whether these CpGs constituted aberrantly methylated regions as defined in Fig. 2, and identified hypomethylation of the *H19/IGF2*:IG-DMR and *IGF2*:Ex9-DMR in the SRS group, hypomethylation of the *MEG3*:TSS-DMR in the TS14 group, and hypermethylation of *MAGEL2*:TSS-DMR, *NDN*:TSS-DMR, *SNRPN*:alt-TSS-DMR, *SNRPN*:Int1-DMR1, *SNRPN*:Int1-DMR2, and *SNURF*:TSS-DMR in the PWS group (Fig. 2). All the identified regions were known iDMRs related to each ID, and no novel aberrantly methylated DMR was found in any group. Thus, there was no hypermethylated or hypomethylated region shared by different ID groups. In addition, there was no novel aberrantly methylated region shared by TS14 and PWS subgroups.

#### Clustering analysis of the methylation pattern in SRS, TS14, and PWS groups

**Fig. 3:**
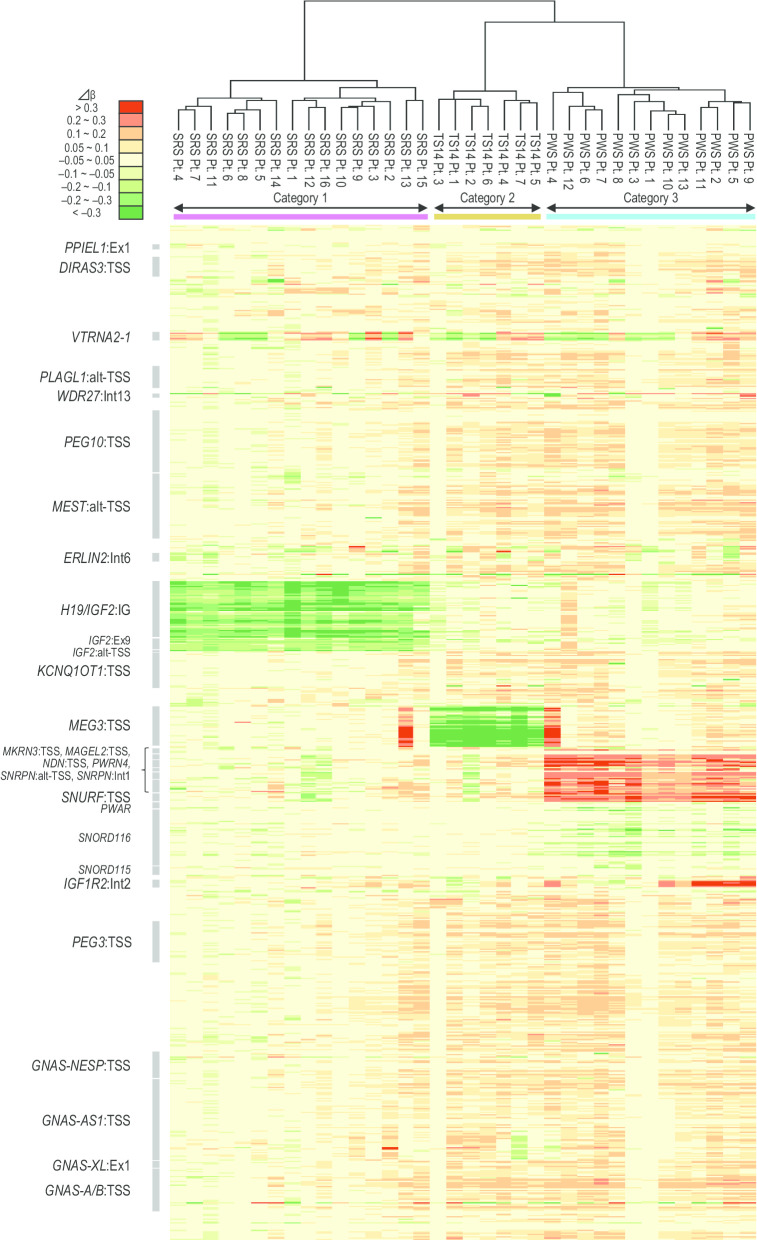
The result of first clustering analysis and heat map of the methylation pattern focusing on the 809 probes at 77 known iDMRs. The patients with SRS, TS14, and PWS are shown in pink, brown, and blue boxes, respectively. The vertical axis indicates the 809 probes at the 77 known iDMRs. The representative iDMRs are shown in gray boxes. *SRS* Silver–Russell syndrome, *TS14* Temple syndrome, *PWS* Prader–Willi syndrome, *iDMRs* imprinting-associated differentially methylated regions

To examine the presence or absence of a characteristic methylation pattern shared by the SRS, TS14, and PWS groups, we performed an unsupervised hierarchal clustering based on ∆*β* of CpGs. We first focused on 809 CpGs at the 77 known iDMRs defined by Monk [28] and Joshi [3]. The 36 patients were classified into three categories based on methylation levels of SRS-, TS14-, and PWS-related iDMRs: (1) category 1, 16 SRS group patients with grossly hypomethylated SRS-related iDMRs on chromosome 11p15.5; (2) category 2, seven TS14 group patients with grossly hypomethylated TS14-related iDMRs on chromosome 14q32.2; and (3) category 3, 13 PWS group patients with grossly hypermethylated PWS-related iDMRs on chromosome 15q11-12 (Fig. 3). Thus, all patients were classified into categories consistent with their original molecular diagnosis.

**Fig. 4:**
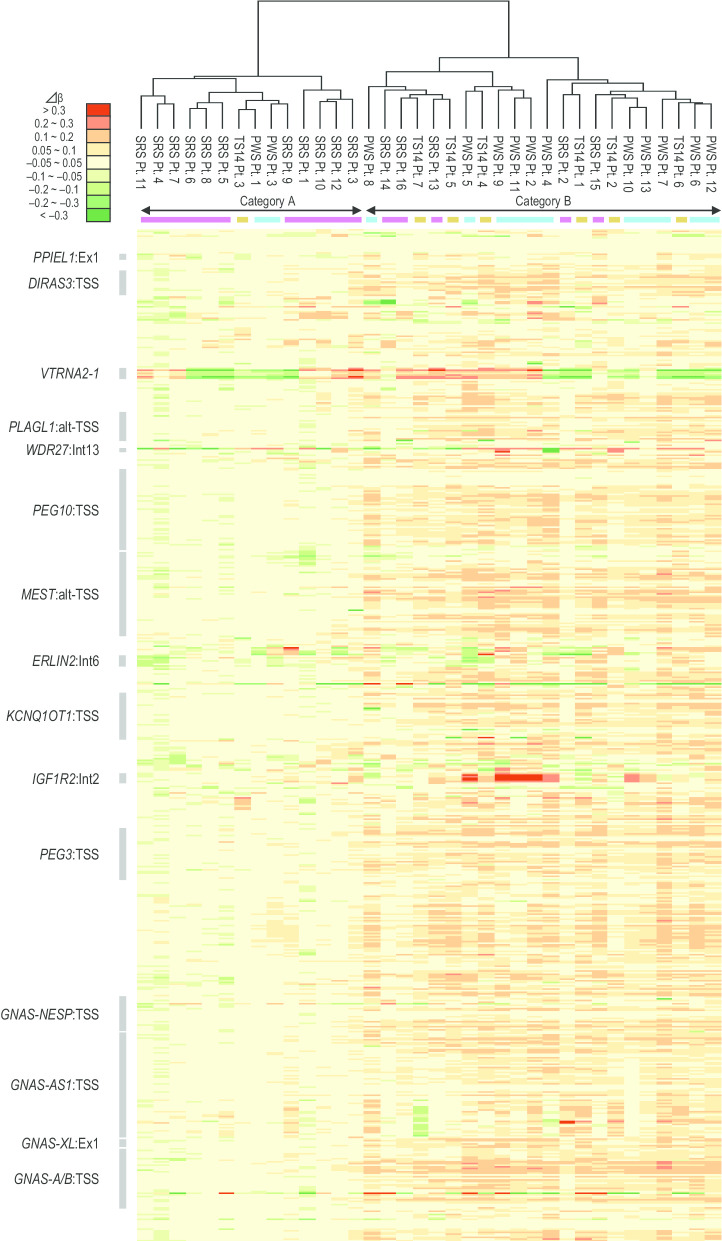
The result of second clustering analysis and heat map of the methylation pattern focusing on the 620 probes at 62 known iDMRs, except for the 15 SRS-, TS14-, and PWS-related iDMRs. The patients with SRS, TS14, and PWS are shown in pink, brown, and blue boxes, respectively. The vertical axis indicates the 620 probes at the 62 known iDMRs. Representative iDMRs are shown in gray boxes. *SRS* Silver–Russell syndrome, *TS14* Temple syndrome, *PWS* Prader–Willi syndrome, *iDMRs* imprinting-associated differentially methylated regions

We next focused on 620 probes at the 62 known iDMRs except for SRS-, TS14-, and PWS-related iDMRs, to perform clustering analysis after excluding the strong effect of these ID-related iDMRs. Consequently, the 36 patients were classified into two categories: (1) category A, 14 patients with grossly normal methylation patterns, and (2) category B, 22 patients with broad and mild hypermethylation patterns (Fig. 4). SRS group patients were primarily classified into category A, whereas five SRS group patients (SRS group Pts. 2 and 13–16) were classified into category B. TS14 group patients were primarily classified into category B, whereas one TS14 group patient (TS14 group Pt. 3) was classified into category A. PWS group patients were primarily classified into category B, whereas two PWS group patients (PWS group Pts. 1 and 3) were classified into category A. However, FDRs of most CpGs within mildly hypermethylated iDMRs were above 0.05 and did not show the statistically significant differences (Additional file 2: Table S2). We performed target methylation analysis using pyrosequencing to validate the methylation levels of four mildly hypermethylated iDMRs (including overlapped CpG sites with HM450k) in category B (Additional file 3: Table S3). However, the methylation levels of four iDMRs analyzed by pyrosequencing were almost consistent between patients in categories A and B. In addition, we examined clinical features of patients (Additional file 1: Table S1) and compared clinical findings between categories A and B. Although SRS group patients classified into category B had more features not characteristic of SRS compared to the patients classified into category A, clinical findings were grossly similar between the two categories of SRS group patients, TS14 group patients, and PWS group patients.

We further focused on 186,927 CpGs within 2000 bp from the transcript start site (TSS) of both imprinted and non-imprinted protein-coding genes. However, we could not identify distinct methylation patterns in SRS, TS14, and PWS groups (data not shown).

## Discussion

This study is the first trial to evaluate the DNA methylation pattern in SRS, TS14, and PWS with overlapping phenotypes using GWMA including 475,000 CpG sites across the human genome.

Our GWMA showed no aberrantly methylated region shared by SRS, TS14, and PWS groups and no novel aberrantly methylated DMR other than iDMR in each group. Unlike our study, previous GWMA in SRS patients [16–18] reported several novel methylation changes in the regions other than *H19/IGF2*:IG-DMR. These studies were intended for only SRS patients and showed inconsistent aberrantly methylated regions due to the differences in bioinformatics analyses and criteria that define aberrantly methylated probes. We identified eight patients with MLID, and three of them had UPD as etiology of the individual ID. Previously, one transient neonatal diabetes mellitus patient with paternal UPD of chromosome 6 and one SRS patient with UPD(7)mat showed MLID [29, 30]. MLID in patients with UPD suggests the presence of the imprinting network interacting among imprinted regions on different chromosomes. The confirmation analysis by pyrosequencing showed normal methylation levels at all additionally affected iDMRs in patients with MLID detected by HM450k. We did not utilize the same control samples in HM450k analysis and pyrosequencing and employed the normal range of pyrosequencing in each iDMR as “the minimum to the maximum” of the results in 50 normal subjects. Furthermore, only some CpG sites examined by pyrosequencing coincide with those analyzed by HM450k. Thus, this discordant result could be caused by the differences in control subjects, normal range values, and examined CpG sites between the two analyses.

Clustering analysis of the methylation patterns focused on the 77 known iDMRs classified the patients into three categories based on the methylation levels of ID-related iDMRs, as expected. This finding underscores the strong effect of ID-related iDMRs for the methylation patterns in SRS, TS14, and PWS patients.

Another clustering analysis of the methylation patterns focused on the 62 known iDMRs, except for SRS-, TS14-, and PWS-related iDMRs, revealed several noteworthy findings. First, patients were classified into two categories, category A with grossly normal methylation patterns and category B with broad and mild hypermethylation patterns. Of note, TS14 and PWS group patients were primarily classified into category B. An expression study by Habib et al. using skin fibroblasts of the patients with TS14 and control subjects suggested the possibility that the increased expression of maternally expressed 14q32.2 non-coding RNAs (ncRNAs), such as *MEG3* and *MEG8*, leads to the decreased gene expression of *IPW* and *SNURF* on 15q11-13 [6]. Although it remains to be clarified whether the 14q32.2 ncRNAs interact directly or indirectly with *IPW* and *SNURF*, the 14q32.2 ncRNAs may have roles in regulating expression of multiple target transcripts in *trans* [31]. In this regard, we speculated that imprinted genes related to mildly hypermethylated iDMRs in this study may mediate the *trans* gene regulation between 14q32.2 and 15q11-13 regions. Second, all patients showed compatible phenotypes of each disease irrespective of their classified category. In addition, SRS group patients classified into category B had more features not characteristic of SRS compared to the patients classified into category A, although we could not perform statistical analysis due to small patient numbers and some missing data. Further studies are required to clarify the association between the clinical phenotypes and the methylation patterns among SRS patients. Lastly, the result of clustering analysis was inconsistent with that of validation analysis using pyrosequencing. As described above, the differences in control subjects, normal range values, and examined CpGs may result in inconsistent results between HM450k and pyrosequencing. Furthermore, because clustering is the partitioning of a data set into categories (clusters) based on some common trait, the results of clustering analysis do not necessarily indicate large differences between categories. Therefore, mild hypermethylation patterns in category B may not be detected by methylation analysis using pyrosequencing.

Our study has some limitations. First, our study focused only on the methylation patterns with genomic DNA from leukocytes and did not perform expression analysis. Indeed, Habib et al. reported decreased expression of *IGF2* in SRS and TS14 patients and decreased expression of *IPW* and *SNURF* in TS14 patients [6]. However, in our GWMA, the methylation patterns of CpGs 2000 bp from TSSs of *IGF2*, *IPW,* and *SNURF* were apparently similar between SRS and TS14 patients and the control subjects. Studies combining GWMA and expression analysis may clarify these matters. Second, we only included SRS patients with *H19*LOM, but not with other molecular etiologies such as UPD(7)mat and duplications and deletions at 11p15, due to their low frequency. Third, we compared only main clinical features of the patients and failed to collect enough clinical data from some patients. Further detailed clinical studies will show the phenotypic differences among patients with different methylation status.

## Conclusions

In this study, we found no methylation signatures shared by SRS, TS14, and PWS groups. Although clustering analysis showed similar mild hypermethylation methylation patterns in TS14 and PWS groups, further study is required to clarify the effect of methylation patterns for overlapping phenotypes.

## Methods

### Subjects

To screen the patients with SRS, TS14, and PWS, we performed methylation analysis using combined bisulfite restriction analysis followed by quantification using Bioanalyzer (Agilent, Santa Clara, CA) [20], pyrosequencing-based methylation analysis [21], or methylation-specific multiplex ligation-dependent probe amplification analysis [32]. The results of these target analyses are shown in Additional file 5: Figure S1. Subsequently, aberrantly methylation of the disease-responsible iDMR(s), hypomethylation of the *H19/IGF2*:IG-DMR in patients with SRS, hypomethylation of the *MEG3*:TSS-DMR in patients with TS14, and hypermethylation of at least one of the PWS-related iDMRs (*SNRPN*:alt-TSS-DMR, *SNRPN*:Int1-DMR, or *SNURF*-TSS-DMR) in patients with PWS were confirmed by GWMA using HM450k. The genetic causes of each ID were examined by microsatellite analysis [33, 34], and custom-built array-based comparative genomic hybridization analysis [35], for the corresponding chromosomes and iDMRs.

### Genome-wide methylation analysis by HM450k

Methylation analysis was carried out on 36 patients with three IDs and 11 healthy child controls using HM450k. The median age was six, nine, two, and two in the SRS group, TS14 group, PWS group, and controls, respectively. HM450k features approximately 475,000 methylation sites across the human genome. Genomic DNA obtained from peripheral blood of patients and controls was treated with bisulfite using the EpiTect plus DNA bisulfite kit (QIAGEN, Hilden, Germany). We subjected bisulfite-treated DNA on array and scanned by the Illumina iScan system.

### Bioinformatics analysis for HM450k data

The workflow of the bioinformatics analysis is shown in Additional file 6: Figure S2. After implementing data processing, we first performed the CH *t*-test for each patient for confirming the abnormal methylation levels of SRS-, TS14-, and PWS-related iDMRs, and for detecting MLID. Subsequently, we utilized (1) “champ.DMP” to reveal aberrantly methylated regions shared by SRS, TS14, and PWS groups/subgroups and (2) unsupervised hierarchal clustering to examine the methylation patterns shared by SRS, TS14, and PWS groups. We excluded probes which show age-related drift [23–25], sex-bias [26], and striking change before/after puberty [27]. All statistical tests were conducted by R version 3.4.1.

### Target analysis using pyrosequencing for validation

To validate the methylation levels of iDMRs in patients with MLID and the results of clustering analysis, we performed targeting analysis using pyrosequencing as previously described [21], except for SRS Pts. 1 and 2 and TS14 Pt. 2 who did not have sufficient sample volumes. Utilized primers are shown in Additional file 4: Table S4. Normal range of the methylation level in each CpG was employed as “the minimum to the maximum” of the results in 50 normal controls.

#### Data preprocessing

We utilized the R package called the Chip Analysis Methylation Pipeline (ChAMP) version 2.8.9 [36]. The data preprocessing consisted of five steps (Additional file 6: Figure S2). First, raw data, IDAT files, were imported with the “champ.load” function to calculate intensity and produce quality control images. We removed probes with detection *P* values above 0.01, probes including fewer than three beads, probes with lower frequency (< 5% of the samples), probes containing SNPs, probes that cross-react to multiple locations, and probes on the sex chromosomes. Second, we used the “champ.QC” function to perform quality control. Third, Beta MIxture Quantile dilation, which is model-based intra-array normalization strategy for HM450k data, was performed with the “champ.norm” function. Fourth, we used the “champ.SVD” function and made singular value decomposition plots to analyze data for potential batch effect. Fifth, batch effect correction was implemented by the Combat algorithm using the empirical Bayes method [37].

#### Methods for confirming methylation levels of disease-related iDMRs in each patient and detecting MLID

To confirm methylation levels of SRS-, TS14-, and PWS-related iDMRs in each patient and examine the presence or absence of MLID, we performed the CH-*t*-test suitable for comparing a single case to a small control group [38] utilizing preprocessed data. For 809 probes at the 77 known iDMRs defined by Monk [28] and Joshi [3], we calculated ∆*β*, the difference between *β* value of each patient and the average *β* value of the control group. We considered a probe as differentially methylated when the absolute value of ∆*β* (|∆*β*|) was above 0.1 and FDR was below 0.05. Based on the criteria defined by Docherty et al. [39], if at least two consecutive probes within an iDMR (including at least four probes) showed differentially methylated levels, the iDMR was defined as aberrantly methylated. The patients who showed aberrantly methylated levels in one or more iDMRs besides their disease-related iDMRs were considered as having MLID. Of note, in patients with UPD(15)mat, we targeted iDMRs other than those on chromosome 15 for the determination of MLID.

#### Methods for detecting an aberrantly methylated regions shared by SRS, TS14, and PWS groups/subgroups

We used the “champ.DMP” function in ChAMP for the calculation of differentially methylated positions (DMPs) between each ID group/subgroup and the control group by a linear model. The DNA methylation level at each probe was converted to *β* values ranging from 0 (completely unmethylated) to 1 (completely methylated). The difference between the average *β* value of each ID group and controls at each probe site (∆*β*) was calculated. We defined the aberrantly methylated probe if its absolute value of ∆*β* (|∆*β*|) was above 0.1 and the FDR using Benjamini–Hochberg was below 0.05. Then, we detected the aberrantly methylated region that satisfied criteria as follows: (1) at least two consecutive probes within iDMRs (including at least four probes) showing aberrant methylation levels or (2) at least three consecutive probes within 2000 bp from TSS showing aberrant methylation levels. We defined these criteria based on the definitions utilized in previous reports [38–40].

#### Methods for clustering analysis of the methylation pattern in SRS, TS14, and PWS groups

We performed unsupervised hierarchal clustering using the Ward method. Clustering was based on ∆*β* which was the difference between the *β* value of each patient and the average *β* value of the control group of probes. Clustering analysis was performed to probes as follows: (1) probes at all known iDMRs [3, 28], (2) probes at known iDMRs, except for 15 SRS-, TS14-, and PWS-related iDMRs [9, 22, 28, 32], and (3) probes within 2000 bp from TSS.

## Supplementary information

**Additional file 1.: Table S1**. Clinical features of patients.

**Additional file 2.: Table S2**. Methylation values of 77 known iDMRs in each patient.

**Additional file 3.: Table S3**. Methylation indices (%) for CpGs within four iDMRs determined by target analysis using pyrosequencing.

**Additional file 4.: Table S4**. Primers utilized in the pyrosequencing analysis.

**Additional file 5.: Figure S1**. The results of target analyses for disease-related iDMRs.

**Additional file 6.: Figure S2**. The workflow of the bioinformatics analysis.

## Data Availability

All data generated or analyzed during this study are available from the corresponding author on reasonable request.

## Abbreviations

ChAMP: Chip Analysis Methylation Pipeline
CH-*t*-test: Crawford–Howell *t* test
DMPs: Differentially methylated positions
FDR: False discovery rate
GWMA: Genome-wide methylation analysis
*H19*LOM: Hypomethylation of the *H19/IGF2*:IG-DMR on chromosome 11p15.5
HM450k: HumanMethylation450 BeadChip
iDMRs: Imprinting-associated differentially methylated regions
IDs: Imprinting disorders
MLID: Multilocus imprinting disturbance
ncRNAs: Non-coding RNAs
NH-CSS: Netchine–Harbison clinical scoring system
PWS: Prader–Willi syndrome
SRS: Silver–Russell syndrome
TS14: Temple syndrome
TSS: Transcription start site
UPD: Uniparental disomy
UPD(7)mat: Maternal uniparental disomy of chromosome 7
UPD(14)mat: Maternal uniparental disomy of chromosome 14
UPD(15)mat: Maternal uniparental disomy of chromosome 15
