# Supplementary material for: Genome-wide methylation analysis in Silver–Russell syndrome, Temple syndrome, and Prader–Willi syndrome
Source: Clin Epigenetics. 2020 Oct 22;12:159. doi: 10.1186/s13148-020-00949-8 (PMC7583213; doi:10.1186/s13148-020-00949-8)
Supplement: Supplementary file 5 — Additional file 5.: Figure S1. The results of target analyses for disease-related iDMRs. [file 13148_2020_949_MOESM5_ESM.pdf]

A

iDMR	CpG	SRS													TS14							PWS		Controls (n=50)
		SRS Pt. 3	SRS Pt. 4	SRS Pt. 5	SRS Pt. 6	SRS Pt. 7	SRS Pt. 9	SRS Pt. 10	SRS Pt. 11	SRS Pt. 12	SRS Pt. 13	SRS Pt. 14	SRS Pt. 15	SRS Pt. 16	TS14 Pt. 1	TS14 Pt. 2	TS14 Pt. 3	TS14 Pt. 4	TS14 Pt. 5	TS14 Pt. 6	TS14 Pt. 7	PWS Pt. 12	PWS Pt. 13	Median (Min ~ Max)
H19/IGF2:IG-DMR (Chr. 11p15.5)	#1	20	27	21	18	28	12	7	16	19	27	10	12	13										48 (37 ~ 60)
	#2	21	32	23	21	28	13	8	18	20	28	11	12	13										50 (39 ~ 64)
	#3	19	24	20	18	27	11	6	15	19	23	9	11	13										46 (36 ~ 57)
	#4	19	25	19	17	24	11	7	15	19	23	9	9	13										45 (36 ~ 55)
MEG3:TSS-DMR (Chr. 14q32.2)	#1														14	2	3	3	2	7	3			52 (43 ~ 56)
	#2														15	4	4	4	3	8	3			55 (52 ~ 65)
	#3														15	3	4	4	4	5	4			37 (32 ~ 55)
	#4														17	5	6	6	5	6	5			60 (44 ~ 74)
	#5														12	1	3	2	2	4	2			36 (26 ~ 47)
SNURF:TSS-DMR (Chr. 15q11.2)	#1																					88	91	55 (50 ~ 60)
	#2																					77	79	49 (44 ~ 54)
	#3																					78	81	51 (46 ~ 57)
	#4																					79	84	52 (47 ~ 57)
	#5																					100	100	63 (58 ~ 68)

B

sites	SRS			Controls (n=40)
	SRS Pt. 1	SRS Pt. 2	SRS Pt. 8	Min ~ Max
*Bsa*B site [*1]	31.5	28		48.1 ~ 64.7
Mwo I site [*2]	28	22.8		50.1 ~ 67.6
Hpy 188I site			13.5	36.2 ~ 58.5
Afl III site			12.2	38.7 ~ 60.0

C

probe	PWS										
	PWS Pt. 1	PWS Pt. 2	PWS Pt. 3	PWS Pt. 4	PWS Pt. 5	PWS Pt. 6	PWS Pt. 7	PWS Pt. 8	PWS Pt. 9	PWS Pt. 10	PWS Pt. 11
NDN probe 04027-L13937 [*1,2]	87.7	83.2	77.0	87.0	85.6	72.3	82.0	100.0	76.2	74.9	90.3
SNRPN probe 11181-L13997	100.0	100.0	86.8	100.0	78.1	100.0	100.0	100.0	100.0	100.0	100.0
SNRPN probe 04106-L13905	60.2	100.0	86.3	100.0	90.0	100.0	98.3	97.0	100.0	100.0	99.0
SNRPN probe 04104-L04294 [*3]	100.0	100.0	83.9	100.0	66.2	100.0	94.2	100.0	100.0	100.0	100.0
SNRPN probe 04103-L02951	99.0	100.0	81.3	100.0	80.1	100.0	76.4	97.7	91.8	100.0	95.8
SNRPN probe 12723-L13798	79.8	67.5	82.7	86.8	63.8	64.4	85.2	88.1	85.6	50.5	64.4

Supplementary Figure S1. A. MIs (%) for CpGs determined by target analysis using pyrosequencing for disease-related iDMRs. Median and normal range are calculated using the results of 50 healthy controls as previously described (Ref.1). The hypomethylated and hypermethylated CpG sites are highlighted with light and dark gray backgrounds, respectively. No CpG site has been overlapped with the array-based methylation analysis with HM450k. **B.** MIs (%) for each site determined by target analysis using Bio-COBRA for the *H19/IGF2*:IG-DMR. A normal range is calculated using the results of 40 healthy controls as previously described (Ref.1, Ref.2). The hypomethylated sites are highlighted with light gray backgrounds. *1 and *2 are CpG sites that have also been examined by the HM450k ([*1]cg04975775, [*2]cg24409677). **C.** MIs (%) for each probe site determined by target analysis using MS-MLPA for PWS-related iDMRs on chromosome 15q11 region. MS-MLPA is performed using the SALSA MS-MLPA ME028kit (MRC-Holland, Amsterdam, Netherlands) based-on the manufacture's protocol (Ref.3). MI is calculated by dividing the normalization constant of each MS-MLPA probe obtained on the digested patient sample by the normalization constant of each MS-MLPA probe obtained on the corresponding undigested sample. *1-3 are probes that have also been examined by the HM450k ([*1]cg12532169, [*2]cg13828758, [*3]cg02125271).

iDMRs, imprinting-associated differentially methylated regions; Chr, chromosome; SRS, Silver-Russell syndrome; TS14, Temple syndrome; PWS, Prader-Willi syndrome; MIs, methylation indices; HM450k, HumanMethylation450 BeadChip; Bio-COBRA, combined bisulfite restriction analysis followed by quantification using Bioanalyzer; MS-MLPA, methylation-specific multiplex ligation-dependent probe amplification.

References:

1. Fuke T, Mizuno S, Nagai T, Hasegawa T, Horikawa R, Miyoshi Y, et al. Molecular and clinical studies in 138 Japanese patients with Silver Russell syndrome. PLoS One. 2013;8:e60105.
2. Yamazawa K, Kagami M, Nagai T, Kondoh T, Onigata K, Maeyama K, et al. Molecular and clinical findings and their correlations in Silver Russell syndrome: implications for a positive role of IGF2 in growth determination and differential imprinting regulation of the IGF2-H19 domain in bodies and placentas. J Mol Med (Berl). 2008;86:1171–81.
3. Matsubara K, Itoh M, Shimizu K, Saito S, Enomoto K, Nakabayashi K, et al. Exploring the unique function of imprinting control centers in the PWS/AS-responsible region: finding from array-based methylation analysis in cases with variously sized microdeletions. Clin Epigenetics. 2019;11:36.
