# Supplementary material for: Genome-wide methylation analysis in Silver–Russell syndrome, Temple syndrome, and Prader–Willi syndrome
Source: Clin Epigenetics. 2020 Oct 22;12:159. doi: 10.1186/s13148-020-00949-8 (PMC7583213; doi:10.1186/s13148-020-00949-8)
Supplement: Supplementary file 6 — Additional file 6.: Figure S2. The workflow of the bioinformatics analysis. [file 13148_2020_949_MOESM6_ESM.pdf]

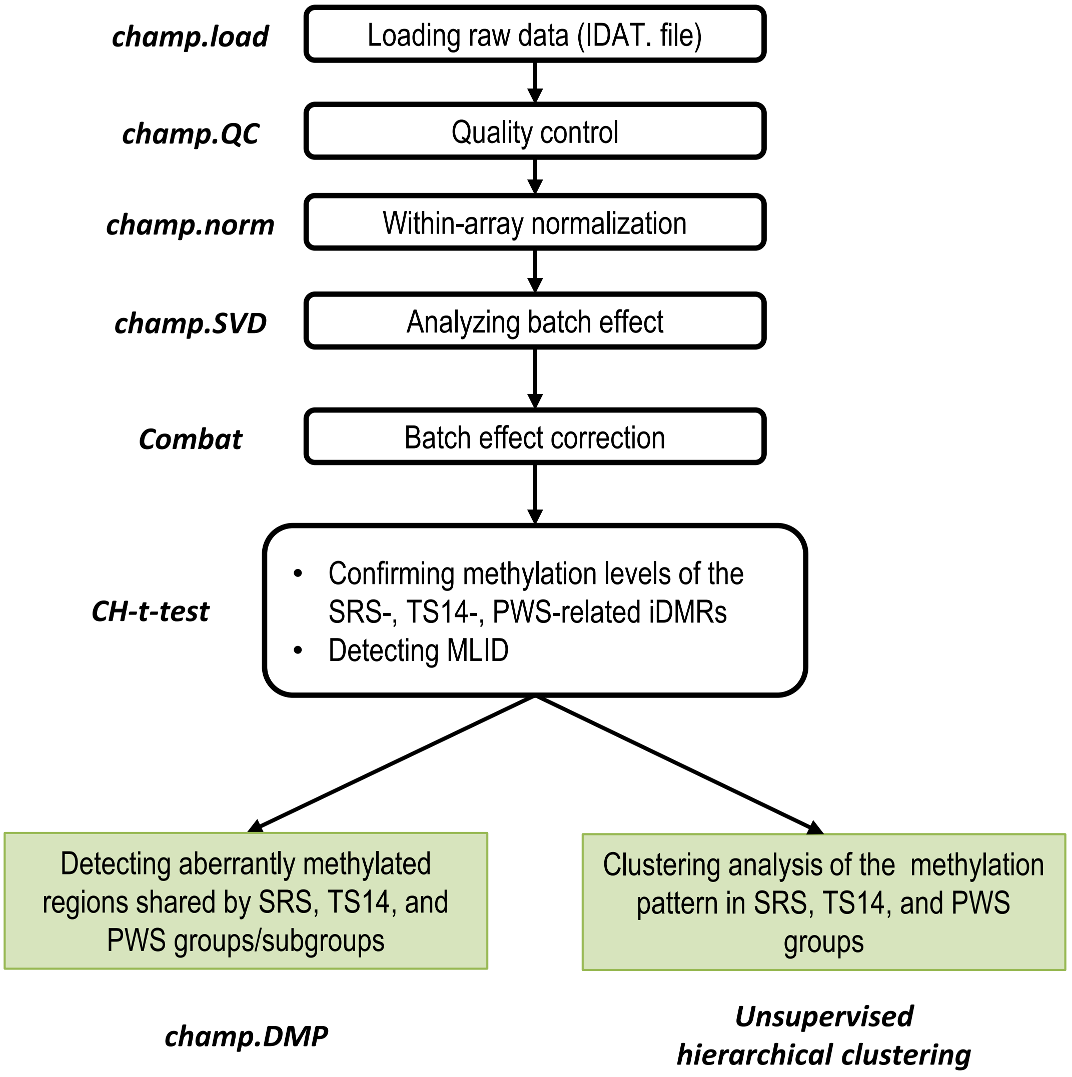

Supplementary Figure S2. The workflow of the bioinformatics analysis. CH-*t*-test, Crawford-Howell *t* test; SRS, Silver-Russell syndrome; TS14, Temple syndrome; PWS, Prader-Willi syndrome; iDMRs, imprinting-associated differentially methylated regions; MLID, multilocus imprinting disturbance.
